# Beyond Microcystins: Cyanobacterial Extracts Induce Cytoskeletal Alterations in Rice Root Cells

**DOI:** 10.3390/ijms21249649

**Published:** 2020-12-17

**Authors:** Dimitris Pappas, Manthos Panou, Ioannis-Dimosthenis S. Adamakis, Spyros Gkelis, Emmanuel Panteris

**Affiliations:** 1 Department of Botany, School of Biology, Aristotle University of Thessaloniki, 54124 Thessaloniki, Greece; mattpano@bio.auth.gr (M.P.); sgkelis@bio.auth.gr (S.G.); 2 Department of Botany, Faculty of Biology, National and Kapodistrian University of Athens, 15784 Athens, Greece; iadamaki@biol.uoa.gr

**Keywords:** bioactive compounds, cyanobacteria, cytoskeleton, F-actin, microcystins, microtubules, *Oryza sativa*, oxidative stress, plant cell

## Abstract

Microcystins (MCs) are cyanobacterial toxins and potent inhibitors of protein phosphatases 1 (PP1) and 2A (PP2A), which are involved in plant cytoskeleton (microtubules and F-actin) organization. Therefore, studies on the toxicity of cyanobacterial products on plant cells have so far been focused on MCs. In this study, we investigated the effects of extracts from 16 (4 MC-producing and 12 non-MC-producing) cyanobacterial strains from several habitats, on various enzymes (PP1, trypsin, elastase), on the plant cytoskeleton and H_2_O_2_ levels in *Oryza sativa* (rice) root cells. Seedling roots were treated for various time periods (1, 12, and 24 h) with aqueous cyanobacterial extracts and underwent either immunostaining for *α*-tubulin or staining of F-actin with fluorescent phalloidin. 2,7-dichlorofluorescein diacetate (DCF-DA) staining was performed for H_2_O_2_ imaging. The enzyme assays confirmed the bioactivity of the extracts of not only MC-rich (MC+), but also MC-devoid (MC−) extracts, which induced major time-dependent alterations on both components of the plant cytoskeleton. These findings suggest that a broad spectrum of bioactive cyanobacterial compounds, apart from MCs or other known cyanotoxins (such as cylindrospermopsin), can affect plants by disrupting the cytoskeleton.

## 1. Introduction

Cyanobacteria are an ancient group of oxygenic photosynthetic prokaryotes, thriving in both aquatic and terrestrial habitats, even under the harshest conditions [1]. Their ability to inhabit numerous diverse environments is also reflected by the plethora of bioactive compounds, which they are able to produce [2]. Among these compounds, a multitude are known to be toxic to other organisms, especially eukaryotes [2], and their potential drug-attributes are an emerging research field (for a review, see [3]).

Τhe most notoriously harmful and well-studied cyanobacterial toxins (also referred to as cyanotoxins), are the microcystins (MCs), harmful to animal cell systems [4], by affecting the cytoskeleton [5]. MCs are monocyclic heptapeptides with two variable amino acids and more than 250 different MC variants exist [2], the most common of which are MC-LR, MC-RR, and MC-YR. MCs are produced by several cyanobacteria, not only of freshwater, but also of marine or terrestrial environments [2], and seem to have functional roles within cyanobacterial cells, such as modulation of specific proteins and protection against oxidative stress [6]. MCs also inhibit serine/threonine protein phosphatases 1 (PP1) and 2A (PP2A) [7,8], which are involved in protein complexes that orchestrate mitotic events [9] and seem to be of great importance for cytoskeleton dynamics, interacting with a wide range of cytoskeleton-associated proteins [10].

Apart from animals, MCs seem to be also toxic for plants [11]. The plant cytoskeleton, i.e., microtubules and F-actin, are among the subcellular components affected by MCs. While microtubules are an established target of MC toxicity in plant cells [12], with MC-derived defects ranging from slight disorientation to total disruption, plant F-actin organization was only recently shown to be negatively affected by MCs [13]. Previous research data on the involvement of PP2A in both microtubule [14] and F-actin [15] organization in *Arabidopsis thaliana* strongly support an association of MC-induced protein phosphatase inhibition with plant cytoskeleton abnormalities. Protein phosphatase (including PP1 and PP2A) homologues related to microtubule regulation have been identified in several model plant species, such as *Arabidopsis thaliana* and *Oryza sativa* [16]. These findings indicate that both cytoskeletal elements are vulnerable to MCs and, thus, deem the plant cytoskeleton a “hot-spot” for studies regarding cyanotoxins and plant cell biology [15].

Oxidative stress is also commonly induced by MCs [17,18,19]. More specifically, hydrogen peroxide (H_2_O_2_) is a reactive oxygen species (ROS) produced during oxidative stress in plants [20], also associated with MC toxicity in plant cells, as increased H_2_O_2_ levels were detected in various plant species, including *Ceratophyllum demersum* [21], *Oryza sativa* [22], and *Nicotiana tabacum* BY-2 cells [23], after exposure to MCs. MC-induced oxidative stress has been held responsible for alterations in cell ultrastructure and chromatin alterations [24,25].

MCs are not the only peptide metabolites produced by cyanobacteria; in fact, cyanopeptides comprise many other—more diverse and far less studied, in comparison to MCs—bioactive molecules, such as cyanopeptolins, aeruginosins, and anabaenopeptins [2,26] and new findings are constantly reported [27]. Interestingly, anabaenopeptins have been experimentally shown to inhibit PP1 activity in vitro [28], raising questions over their ability to induce disruption of the cytoskeleton. In her recent review, Janssen [26] underlines the significance of using extracts from cyanobacterial strains as reference, in order to simulate field exposure to cyanopeptides. Indeed, crude strain extracts are naturally occurring mixtures of bioactive compounds [27,29], closely matching real-life exposure. Accordingly, extracts have been extensively used to study the adverse effects of cyanobacterial compounds on plant cell physiology [30,31,32]. Among plant species, rice (*Oryza sativa*) is an ideal experimental material for such research, due to its importance for food production [33] and the fact that it is usually cultivated in fields flooded with water, potentially containing cyanobacteria and their metabolic products.

In this context, we carried out an extensive screening of the toxic effects of a variety of MC-rich (MC+) and MC-devoid (MC−) cyanobacterial extracts on rice roots, in order to broaden the data concerning the potential adverse toxic effects of cyanobacteria on plant cells. Cyanotoxins, such as MCs and cylindrospermopsin, are known to be uptaken by the roots of various crop species [34,35], so root cells are expected to exhibit possible cytoskeletal alterations due to treatment with the extracts. Interestingly enough, extracts from strains not yet reported to produce any cyanotoxins, appear to exert severe effects on rice cytoskeleton. These observations reveal that cyanobacteria could negatively affect plants through a broad “arsenal” of bioactive compounds.

## 2. Results

### 2.1. Enzyme Inhibition

In order to preliminarily assess the inhibitory potential of the cyanobacterial extracts used in this study, we examined the inhibition of three enzymes, PP1, trypsin, and elastase, typically included in tests for cyanobacterial toxicity [28,36] (Table 1). Only MC+ extracts (1410, 2410, and 1614) inhibited PP1 activity. Trypsin was inhibited only by the 1614 extract. All of the extracts tested, except for 0717, were found to inhibit elastase activity. Accordingly, toxicity due to cyanotoxins is expected to occur under treatment with these extracts.

### 2.2. Effects on F-actin

#### 2.2.1. Morphological Alterations

**Figure 1:**
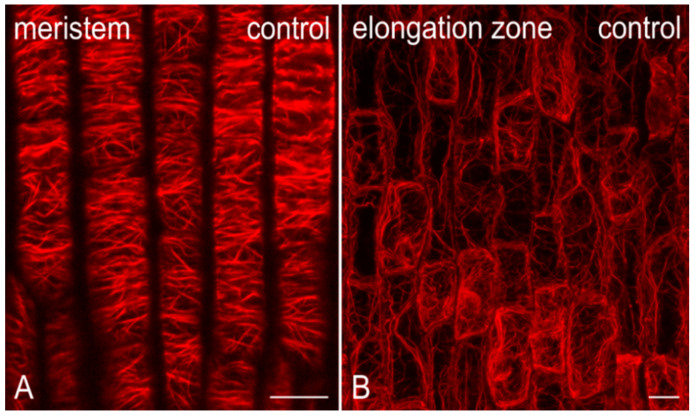
Single cortical confocal laser scanning microscope (CLSM) section (**A**) and maximum intensity projection of serial CLSM sections (**B**) of control *Oryza sativa* root, depicting protodermal cells in the apical meristem (**A**) and epidermal cells in the elongation zone (**B**), after F-actin staining. Root tips in both images point towards the bottom of the page. Control meristematic cells exhibit abundant fine cortical actin filaments, with a dominant transverse orientation (**A**), while elongating cells typically exhibit large, longitudinal subcortical F-actin cables (**B**). Scale bars: 10 μm.

**Figure 2:**
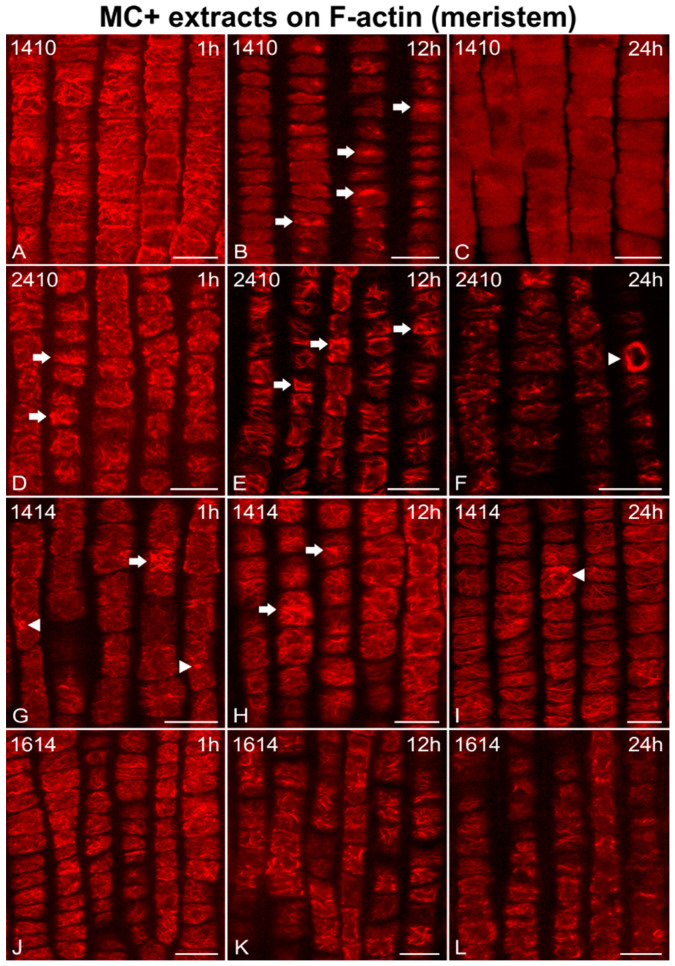
Single cortical CLSM sections of *Oryza sativa* root protodermal cells stained for F-actin, after treatment with MC+ extracts from various MC-producing strains: *Microcystis flos-aquae* TAU-MAC 1410 (**A**–**C**), *Microcystis* sp. TAU-MAC 2410 (**D**–**F**), *Raphidiopsis raciborskii* TAU-MAC 1414 (**G**–**I**), and *Trichormus variabilis* TAU-MAC 1614 (**J**–**L**). Root tips in all images point towards the bottom of the page. After 1 h of treatment, actin filaments were disoriented and branched (**A**; cf. Figure 1A) and tended to form bundles (arrows in **D**,**G**) or even rings (arrowheads in **G**). After 12 h, bundling (arrows in **B**,**E**,**H**) and disorientation (**E**,**H**,**K**) appeared intensified. After 24 h, the F-actin network deteriorated significantly (**F**,**L**) and appeared disoriented (**I**), or even collapsed (**C**). Circular actin aggregates (arrowhead in **F**) and ring-shaped conformations (arrowhead in **I**) could also be detected. Scale bars: 10 μm.

**Figure 3:**
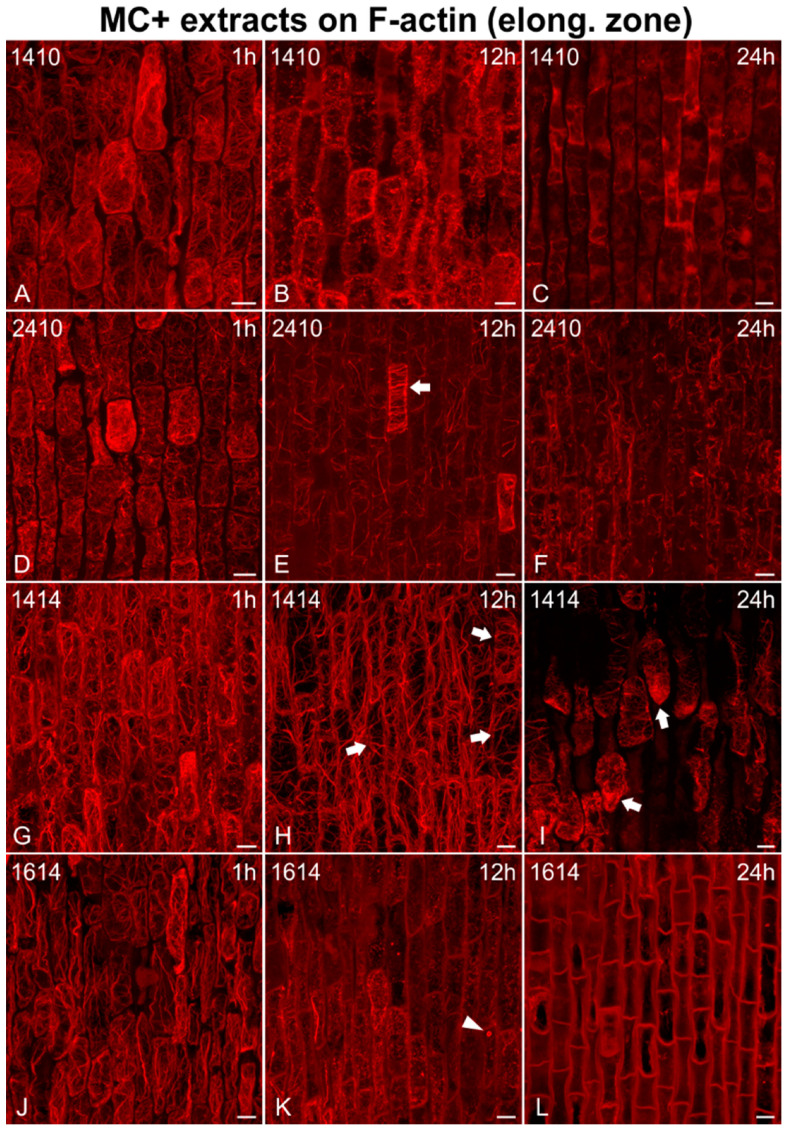
Maximum intensity projections of serial CLSM sections of *Oryza sativa* epidermal cells in the root elongation zone, stained for F-actin, after treatment with MC+ extracts from various MC-producing strains: *Microcystis flos-aquae* TAU-MAC 1410 (**A**–**C**), *Microcystis* sp. TAU-MAC 2410 (**D**–**F**), *Raphidiopsis raciborskii* TAU-MAC 1414 (**G**–**I**), and *Trichormus variabilis* TAU-MAC 1614 (**J**–**L**). Root tips in all images point towards the bottom of the page. After 1 h of treatment (**A**,**D**,**G**,**J**) F-actin network appeared almost unaffected, compared to the control (Figure 1B). After 12 h, actin cables appeared highly affected, being either fragmented (**B**), disoriented (arrows in **E**,**H**), or scarce (**K**, note also the actin ring indicated by arrowhead). After 24 h, F-actin either disappeared (**C**,**L**) or was fragmented (**F**). Actin aggregates could also be detected (arrows in **I**). Scale bars: 10 μm.

Microfilaments were abundant in control root cells (Figure 1), of the apical meristem (Figure 1A) and the elongation zone (Figure 1B), while cells treated with MC+ extracts (from MC-producing strains; 1410, 2410, 1414, 1614) exhibited time-dependent alterations of the F-actin cytoskeleton (Figure 2 and Figure 3). After just 1 h of treatment, cortical actin filaments in meristematic root cells appeared to be disoriented and branched (Figure 2A,J) or even bundled (arrows in Figure 2D,G), sometimes exhibiting ring-shaped F-actin conformations (arrowheads in Figure 2G). After 12 h, cortical microfilaments, were heavily disoriented (Figure 2E,H,K) and bundled (arrows in Figure 2B,E,H). In 1410-treated root cells, except for bundles, no discernable actin filaments could be observed after 12 h (Figure 2B), while F-actin was almost absent after 24 h (Figure 2C). Treatment with the other MC+ extracts for 24 h led to various F-actin defects, ranging from disorientation (Figure 2I) and the appearance of actin circular conformations/rings (arrowheads in Figure 2F,I), to severe deterioration of the F-actin network integrity (Figure 2L).

Cells in the root elongation zone were also affected (Figure 3). Longitudinal F-actin cables, a common feature of untreated cells in the elongation zone (Figure 1B), could still be observed after 1 h of treatment (Figure 3A,D,G,J). However, after 12 h, these cables appeared significantly altered in various ways, being either disoriented (Figure 3E,H), scarce (Figure 3Κ), or even absent, in which case F-actin was severely damaged and fragmented (Figure 3B). Eventually, after 24 h, actin filaments either disappeared completely (Figure 3C,L), or were scarce and disoriented, wherever present (Figure 3F,I). Actin aggregates were also detected (arrows in Figure 3I).

**Figure 4:**
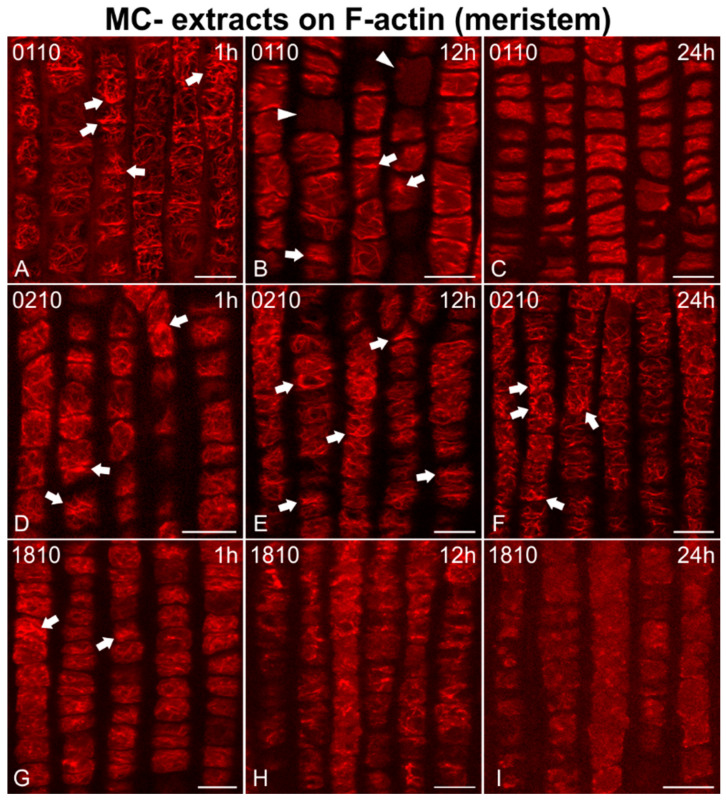
Single cortical CLSM sections of *Oryza sativa* root protodermal cells stained for F-actin, after treatment with MC− extracts, from various non-MC-producing strains: *Jaaginema* sp. TAU-MAC 0110 (**A**–**C**), *Jaaginema* sp. TAU-MAC 0210 (**D**–**F**), and *Microcystis viridis* TAU-MAC 1810 (**G**–**I**). Root tips in all images point towards the bottom of the page. The 1 h of treatment led to disorientation (**A**) and bundling (arrows in **A**,**D**,**G**) of F-actin. After 12 h, disorientation and bundling effects were more intense (arrows in **B**,**E**) or F-actin was fragmented (**H**). In some cases, cells devoid of F-actin could be observed (arrowheads in **B**). After 24 h, F-actin was degraded (**C**) and disoriented/bundled (arrows in **F**), or even disappeared (**I**). Scale bars: 10 μm.

**Figure 5:**
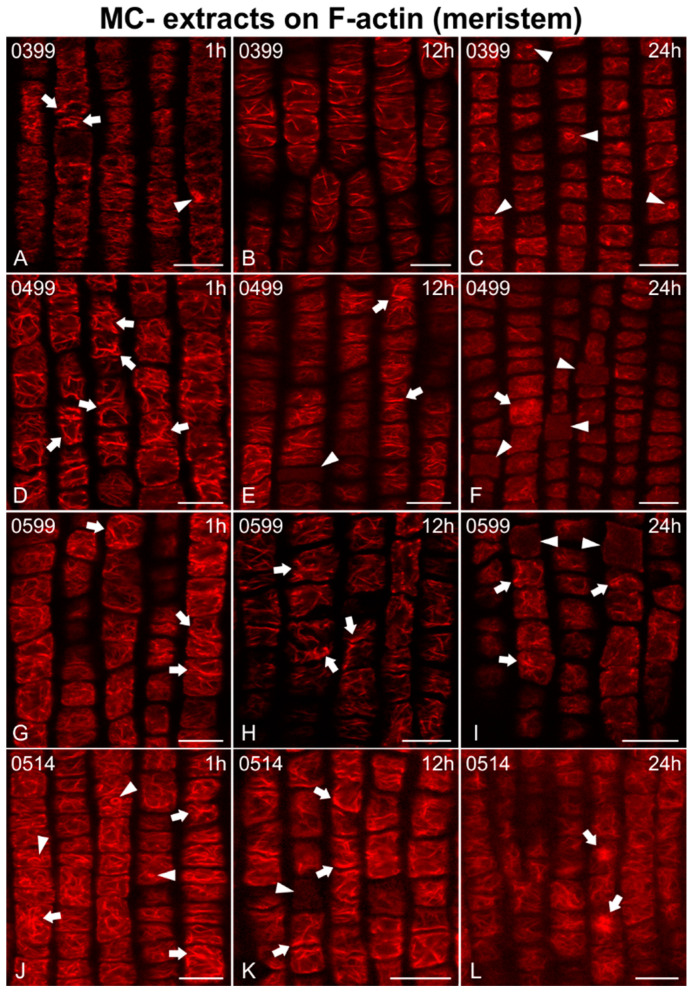
Single cortical CLSM sections of *Oryza sativa* root protodermal cells stained for F-actin, after treatment with MC− extracts from various non-MC-producing strains: *Calothrix epiphytica* TAU-MAC 0399 (**A**–**C**), *Synechococcus* sp. TAU-MAC 0499 (**D**–**F**), *Chlorogloeopsis fritschii* TAU-MAC 0599 (**G**–**I**), and *Planktothrix agardhii* TAU-MAC 1614 (**J**–**L**). Root tips in all images point towards the bottom of the page. Cells treated for 1 h exhibited disoriented/branched and bundled (arrows in **A**,**D**,**G**,**J**) cortical actin filaments, along with actin aggregates (arrowhead in **A**) and actin rings (arrowheads in **J**) in some cases. Scarce disoriented (**B**,**H**) and bundled (arrows in **E**,**H**,**K**) were also reported after 12 h, while, occasionally, cells devoid of F-actin (arrowheads in **E**,**K**) could be observed. After 24 h, actin filaments were weak and scarce (**C**,**F**,**I**) or heavily disoriented (**L**) with actin aggregates (arrows in **F**,**L**) and bundles (arrows in **I**), while actin rings (arrowheads in **C**) or cells almost without detectable F-actin (arrowheads in **F**,**I**) could be noticed. Scale bars: 10 μm.

**Figure 6:**
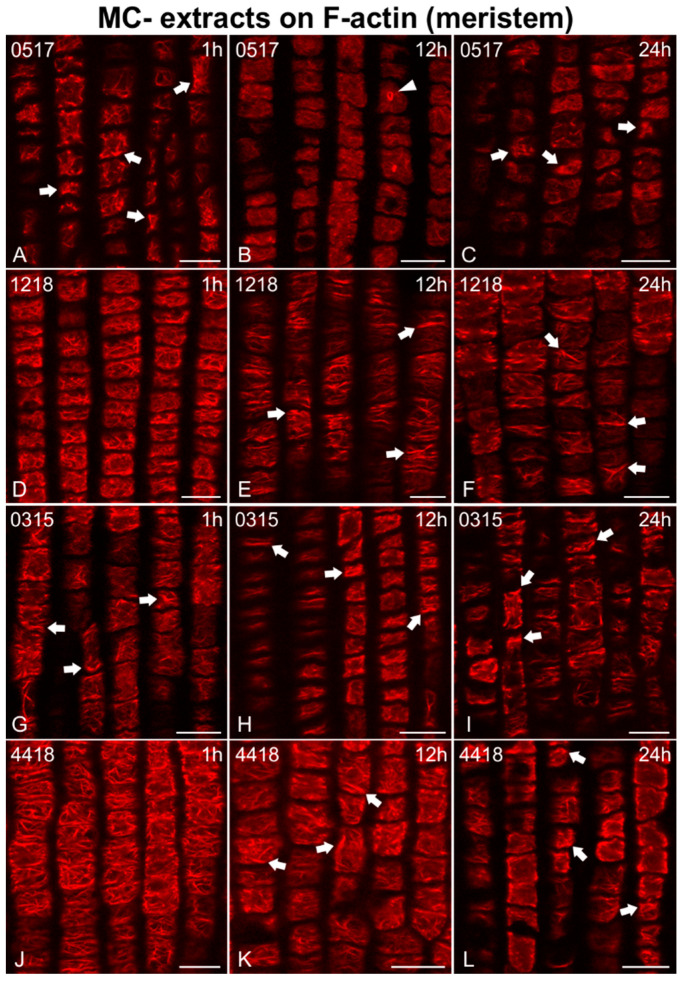
Single cortical CLSM sections of *Oryza sativa* root protodermal cells stained for F-actin, after treatment with MC− extracts from various non-MC-producing strains: *Phormidium* sp. TAU-MAC 0517 (**A**–**C**), *Scytonema* sp. TAU-MAC 1218 (**D**–**F**), *Nostoc oryzae* TAU-MAC 0315 (**G**–**I**), and *Lyngbya* sp. TAU-MAC 4418 (**J**–**L**). Root tips in all images point towards the bottom of the page. After 1 h of exposure to the extracts, affected cells exhibited bundled (arrows in **A**,**G**) and branched cortical actin filaments (**D**,**J**). After 12 h, the F-actin network deteriorated (**B**,**E**,**H**) and bundles could be observed (arrows in **E**,**H**,**K**), with occasional actin rings (arrowhead in **B**). After 24 h, actin filaments were scarce and bundled (arrows in **F**,**I**,**L**) or weak, with actin aggregates being detected (arrows in **C**). Scale bars: 10 μm.

In addition, F-actin was adversely affected in root meristematic cells after treatment with MC− extracts (from strains not producing MCs; Figure 4, Figure 5 and Figure 6). Disorientation/branching of cortical actin filaments (Figure 4A, Figure 5G and Figure 6D,J), bundling (Figure 4D,G and Figure 5A), or a combination of these effects (Figure 5D,J and Figure 6A,G) were commonly observed in affected cells after only 1 h of treatment. Actin rings were also detected in some cases (arrowheads in Figure 5J). These effects persisted or were intensified after 12 h (Figure 4B,E, Figure 5B,E,H,K and Figure 6E,H,K), while loss of actin network integrity (Figure 4H and Figure 6B), ring-shaped conformations (arrowhead in Figure 6B), and even cells devoid of F-actin (arrowheads in Figure 4B and Figure 5E,K) could be observed. After 24 h, actin filaments were either weak and scarce (Figure 4C, Figure 5F,I and Figure 6L) occasionally forming rings (arrowheads in Figure 5C), heavily bundled (Figure 4F, Figure 5L and Figure 6F,I,L), or eventually disappearing (Figure 4I, arrowheads in Figure 5F,I).

**Figure 7:**
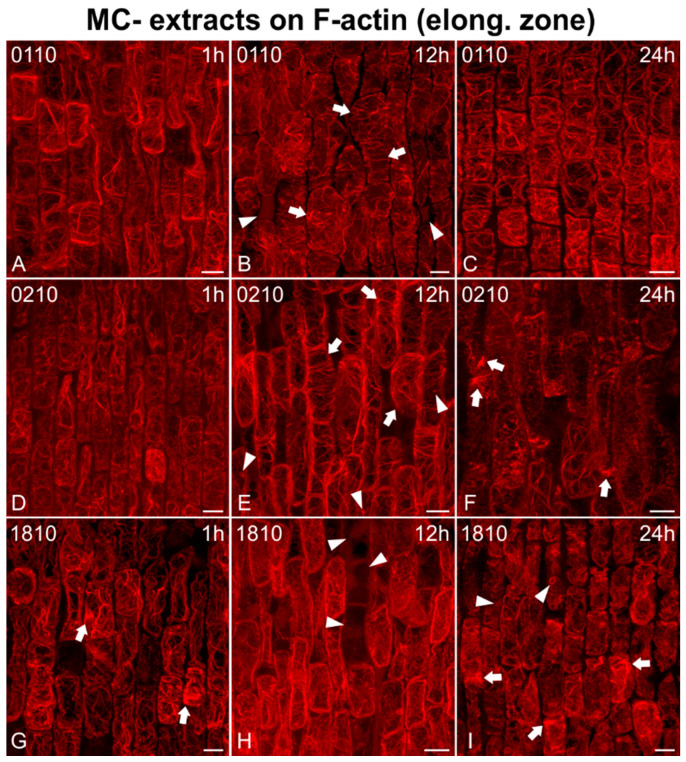
Maximum intensity projections of serial CLSM sections of *Oryza sativa* epidermal cells in the root elongation zone, stained for F-actin, after treatment with MC− extracts from various non-MC-producing strains: *Jaaginema* sp. TAU-MAC 0110 (**A**–**C**), *Jaaginema* sp. TAU-MAC 0210 (**D**–**F**), and *Microcystis viridis* TAU-MAC 1810 (**G**–**I**). Root tips in all images point towards the bottom of the page. Actin cables were not significantly affected after 1 h of treatment (**A**,**D**), except for some actin aggregates (arrows in **G**). After 12 h, F-actin disorientation was visible (arrows in **B**,**E**), along with actin rings (arrowheads in **E**) and cells devoid of F-actin (arrowheads in **B**,**H**). After 24 h, actin rings were still detectable (arrowheads in **I**), as well as disorientation (**C**) and actin aggregates (arrows in **F**,**I**). Scale bars: 10 μm.

**Figure 8:**
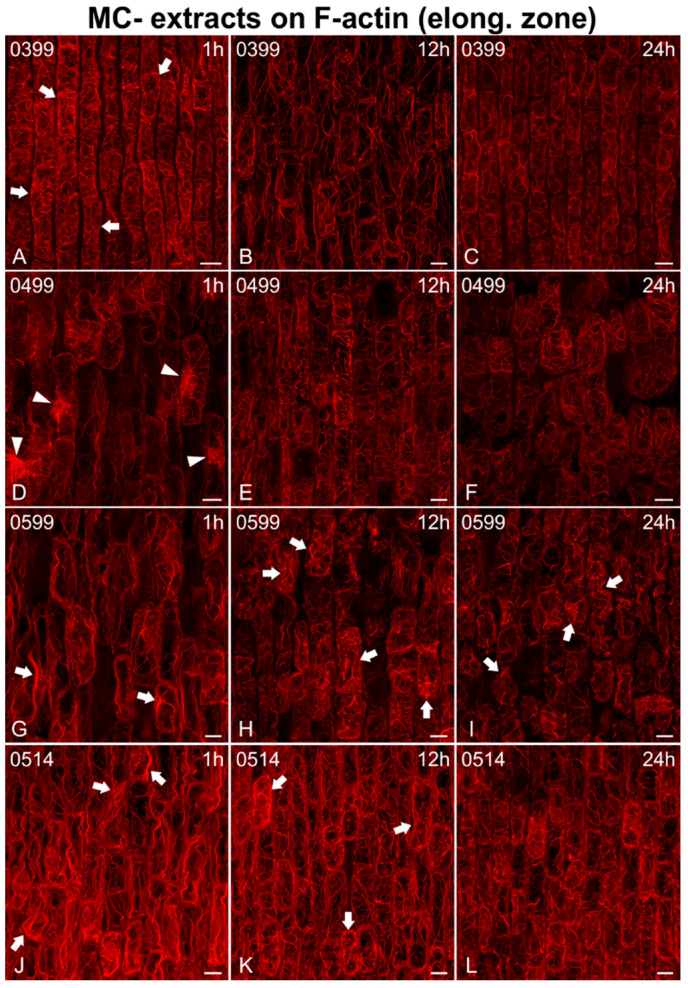
Maximum intensity projections of serial CLSM sections of *Oryza sativa* epidermal cells in the root elongation zone, stained for F-actin, after treatment with MC− extracts from various non-MC-producing strains: *Calothrix epiphytica* TAU-MAC 0399 (**A**–**C**), *Synechococcus* sp. TAU-MAC 0499 (**D**–**F**), *Chlorogloeopsis fritschii* TAU-MAC 0599 (**G**–**I**), and *Planktothrix agardhii* TAU-MAC 1614 (**J**–**L**). Root tips in all images point towards the bottom of the page. After 1 h of treatment, F-actin was disoriented (arrows in **A**) or exhibited actin aggregates (arrowheads in **D**) and minor bundling effects (arrows in **G**,**J**). Diminishing of the F-actin network (**B**,**E**) and bundling of actin cables (arrows in **H**,**K**) were visible after 12 h. F-actin was further degraded after 24 h (**C**,**F**,**I**,**L**), with some bundling effects observed (arrows in **I**). Scale bars: 10 μm.

**Figure 9:**
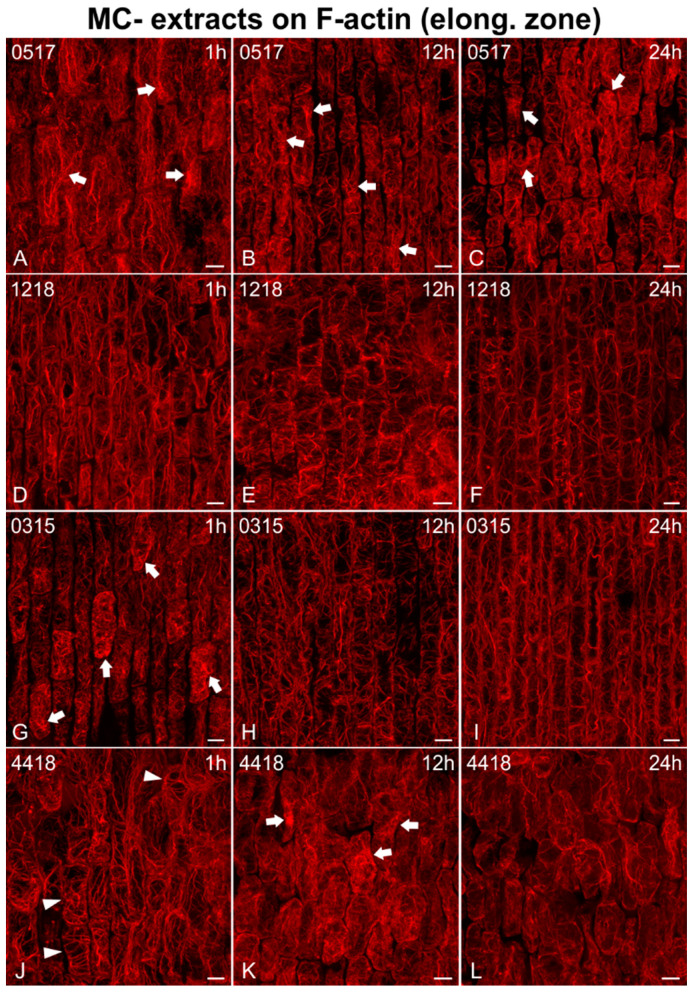
Maximum intensity projections of serial CLSM sections of *Oryza sativa* epidermal cells in the root elongation zone, stained for F-actin, after treatment with MC− extracts from various non-MC-producing strains: *Phormidium* sp. TAU-MAC 0517 (**A**–**C**), *Scytonema* sp. TAU-MAC 1218 (**D**–**F**), *Nostoc oryzae* TAU-MAC 0315 (**G**–**I**), and *Lyngbya* sp. TAU-MAC 4418 (**J**–**L**). Root tips in all images point towards the bottom of the page. Cells treated for 1 h either exhibited minor bundling of F-actin (arrows in **A**,**G**) or retained a control-like state (**D**,**J**; cf. Figure 1B), with occasional minor disorientation effects (arrowheads in **J**). After 12 h, bundling effects remained (arrows in **B**) or progressed to the formation of actin aggregates (arrows in **K**), while disorientation of actin cables was also visible (**E**,**H**). Actin aggregates (arrows in **C**) and F-actin deterioration (**F**,**I**,**L**) were common alterations observed after 24 h of treatment. Scale bars: 10 μm.

As for the elongation zone (Figure 7, Figure 8 and Figure 9), short treatments (1 h) with MC− extracts (from non-MC-producing strains) did not greatly affect F-actin in elongating cells (Figure 7A,D and Figure 9D,J), except for some bundling/aggregates (arrows in Figure 7G, Figure 8D,G,J and Figure 9A,G,J) or disorientation effects (transverse actin cables instead of longitudinal) noticed (arrows in Figure 8A, arrowheads in Figure 9J). After 12 h, disorientation of actin cables (Figure 7B,E and Figure 9E,H) and F-actin bundling/aggregates (arrows in Figure 8H,K and Figure 9B,K) were common effects, along with F-actin diminishing (Figure 8B,E) and, in some cases, actin rings (arrowheads in Figure 7E) and cells devoid of F-actin (arrowheads in Figure 7B,H). After 24 h, the F-actin network was heavily disoriented, bundled, or degraded (Figure 7C,F,I, Figure 8C,F,I,L and Figure 9C,F,I,L). Actin rings were also detected (arrowheads in Figure 7I).

#### 2.2.2. F-actin Fluorescence Intensity Measurements

**Figure 10:**
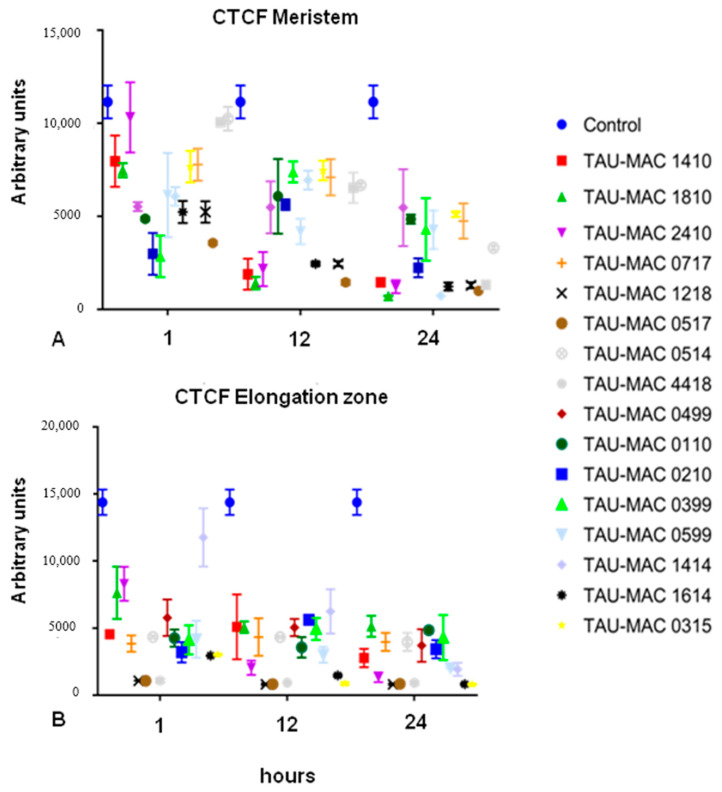
Graphs illustrating the corrected total cell fluorescence (CTCF) intensity measurements of untreated (control) and extract-treated root meristem (**A**) and elongation zone cells (**B**). Note that fluorescence intensity drops significantly even upon 1 h of treatment, especially in the elongating root cells.

The detrimental effects of the cyanobacterial extracts on F-actin in rice root cells were further confirmed by measurements of the corrected total cell fluorescence (CTCF; Figure 10). In both meristematic (Figure 10A) and elongation zone root cells (Figure 10B), CTCF dropped upon treatment with each cyanobacterial extract, readily from 1 h of the extract application. The exposed elongating root cells exhibited a pronounced fluorescence intensity drop, compared to the meristematic cells (Figure 10B; cf. Figure 10A). CTCF measurements drop observed in all extract treatments, showed a statistical significance, set at *p* < 0.05.

### 2.3. Effects on Microtubules and Chromatin

**Figure 11:**
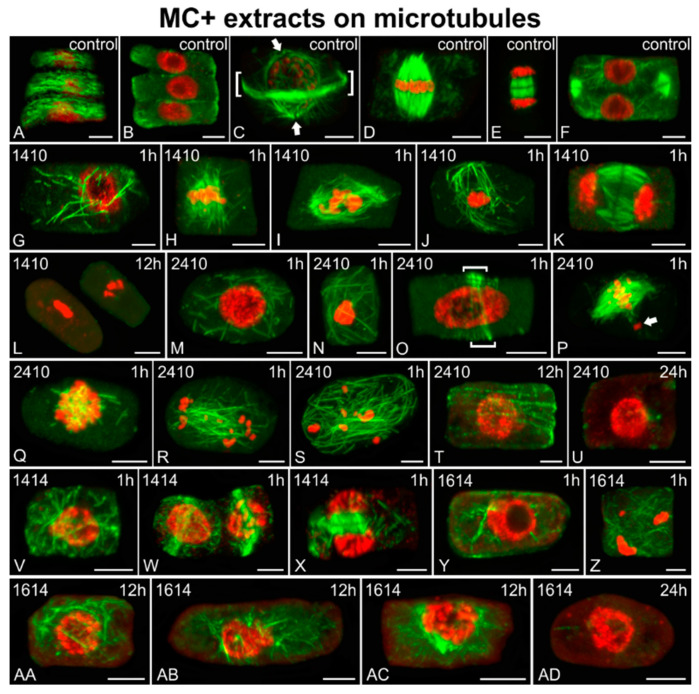
Single cortical (**A**), single central (**B**,**K**,**X**,**Y**), and maximum intensity projections of serial CLSM sections (**C**–**J**,**L**–**W**,**Z**–**AD**) of *Oryza sativa* root meristematic cells, after α-tubulin immunostaining (green) and DNA staining with DAPI (pseudo-coloration in red). Cells depicted are either control (**A**–**F**) or treated for various time periods (indicated on each image) with MC+ extracts from MC-producing cyanobacterial strains: *Microcystis flos-aquae* TAU-MAC 1410 (**G**–**L**), *Microcystis* sp. TAU-MAC 2410 (**M**–**U**), *Raphidiopsis raciborskii* TAU-MAC 1414 (**V**–**X**), and *Trichormus variabilis* TAU-MAC 1614 (**Y**–**AD**). Images (**A**) and (**B**) depict the same cells at cortical and central sections. (**A**–**F**). Control cells at interphase exhibit dense, transverse cortical microtubules (**A**) and no endoplasmic microtubules (**B**). Preprophase/prophase cells exhibit the typical preprophase band (PPB) of microtubules (brackets in **C**), as well as perinuclear microtubules converging on two distinctive poles (arrows in **C**). Mitotic spindles with aligned chromosomes at the equator can be observed in metaphase cells (**D**) and control cytokinetic cells exhibit typical phragmoplasts (early stage in **E** and later stage in **F**). **1410**: After 1 h of treatment, scarce and disoriented interphase microtubules were observed (**G**). Abnormal mitotic spindles were detected (**H**,**I**), along with cells exhibiting misoriented microtubules and abnormal chromatin condensation (**J**). Abnormally elongated phragmoplast microtubules were observed in cytokinetic cells (**K**). The 12 h of treatment led to the complete disappearance of microtubules, with chromatin also appearing highly condensed (**L**). **2410**: After 1 h, short and disorganized microtubules were observed not only in interphase cells (**M**), but also in cells with uncommon chromatin condensation (**N**). Preprophase cells lacking perinuclear microtubules (**O**) were spotted, along with distorted mitotic spindles (note the misaligned chromosome outside the spindle, pointed with arrow in **P**). Affected cells of undefined chromatin state exhibited extremely short (**Q**) or misplaced and disoriented microtubules (**R**,**S**). After 12 h, only interphase cells with a sparse microtubule network were encountered (**T**), which eventually collapsed after 24 h (**U**). **1414**: After 1 h of treatment, disorientation was common for both interphase (**V**) and perinuclear microtubules in preprophase cells (**W**), as well as phragmoplast microtubules in affected cytokinetic cells (**X**). Harsher effects were not recorded after longer treatments. **1614**: After 1 h of treatment, affected cells exhibited scarce and misoriented microtubules (**Y**) and, in some cases, abnormally condensed and scattered chromatin (**Z**). After 12 h, disorientation of the microtubule network could still be observed in interphase (**AA**), also with abundant short perinuclear microtubules (**AB**). Preprophase cells without a PPB were recorded (**AC**). After 24 h, almost no tubulin polymers could be detected (**AD**). Scale bars: 5 μm.

Microtubules were severely affected by MC+ extracts (Figure 11), compared to the control (Figure 11A–F). *Microcystis* (1410 and 2410) extracts exhibited their effects on root meristematic cells starting from 1 h of exposure. Misoriented (Figure 11G,N,S) or even fractured (Figure 11M,Q) microtubules, deformed mitotic spindles (Figure 11H–J,P,R; cf. Figure 11D) and phragmoplasts (Figure 11K; cf. Figure 11F), absence of perinuclear microtubules in preprophase cells (Figure 11O; cf. Figure 11C), and abnormal condensation (Figure 11J,N) or dispersal (Figure 11R,S) of chromatin were observed in affected cells after only 1 h of treatment. After 12 h, root cells affected by the 1410 extract exhibited no microtubules, as well as abnormally condensed chromatin (Figure 11L), while cells treated with the 2410 extract exhibited scarce cortical microtubules (Figure 11T; cf. Figure 11A), which eventually disappeared after 24 h (Figure 11U). Extracts from other MC+ strains (1414 and 1614) exhibited slighter effects on rice root cells than the *Microcystis* extracts. After short-term treatments (1 h), 1414-treated root cells exhibited mainly microtubule disorientation in various cell cycle stages, such as cortical microtubules in interphase cells (Figure 11V; cf. Figure 11A), perinuclear microtubules in preprophase cells (Figure 11W; cf. Figure 11C) and phragmoplast microtubules (Figure 11X; cf. Figure 11E), without further disturbance after longer treatments. Short-term (1 h) treatment with the 1614 extract also disturbed the microtubule network, leading to the prevalence of endoplasmic microtubules in affected cells (Figure 11Y; cf. Figure 11B) and, in some cases, chromatin dispersal (Figure 11Z). After 12 h, disoriented cortical (Figure 11AA; cf. Figure 11A) and endoplasmic microtubules (Figure 11AB; cf. Figure 11B), along with preprophase cells lacking preprophase band (PPB) (Figure 11AC; cf. Figure 11C), were observed. Eventually, after 24 h treatment with the 1614 extract, microtubules disappeared almost totally (Figure 11AD).

**Figure 12:**
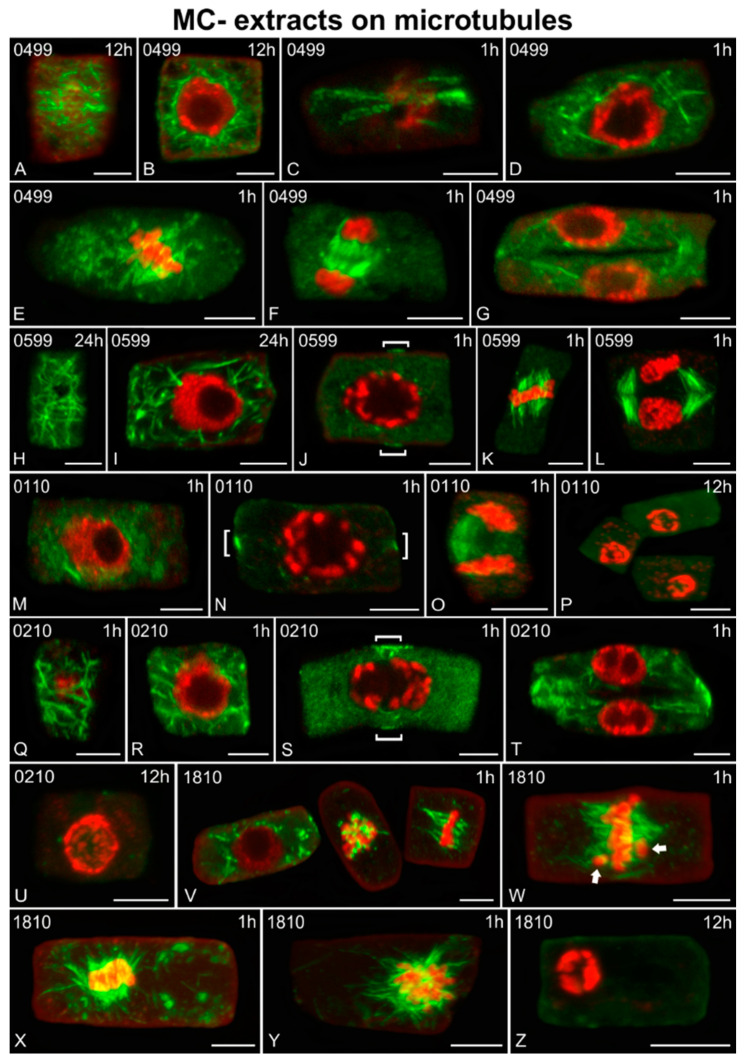
Single cortical (**A**,**C**,**H**,**Q**), single central (**B**,**D**,**F**,**G**,**I**,**J**,**M**–**O**,**S**,**T**,**V**), and maximum intensity projections of serial (**E**,**K**,**L**,**P**,**U**,**W**–**Z**) CLSM sections of *Oryza sativa* root meristematic cells, after α-tubulin immunostaining (green) and DNA staining with DAPI (pseudo-coloration in red). The cells depicted are from roots treated for various time periods (indicated on each image) with MC− extracts from non-MC-producing cyanobacterial strains: *Synechococcus* sp. TAU-MAC 0499 (**A**–**G**), *Chlorogloeopsis fritschii* TAU-MAC 0599 (**H**–**L**), *Jaaginema* sp. TAU-MAC 0110 (**M**–**P**), *Jaaginema* sp. TAU-MAC 0210 (**Q**–**U**), and *Microcystis* sp. TAU-MAC 1810 (**V**–**Z**). Images (**A**–**D**) and (**Q**,**R**) depict the same cells at cortical and central sections. **0499**: Affected interphase cells (**A**–**D**) exhibited diminished cortical (**A**,**C**) and prevalent perinuclear (**B**,**D**) microtubules. In affected mitotic cells, short dispersed spindle microtubules (**E**), almost normal early phragmoplasts (**F**), as well as disorganized advanced phragmoplasts (**G**, though some long microtubules persist) with thick floating cell plates, were observed. **0599**: Affected cells exhibited disoriented cortical (**H**) and many perinuclear and endoplasmic microtubules (**I**) at interphase, faint PPB and scarce perinuclear microtubules at preprophase (**J**, brackets indicate PPB), short spindle microtubules at metaphase (**K**) and long phragmoplast microtubules at cytokinesis (**L**). **0110**: After 1 h of treatment, endoplasmic tubulin aggregations at interphase (**M**), absence of perinuclear microtubules at preprophase (**N**, brackets indicate PPB) and unilateral, long phragmoplast microtubules at cytokinesis (**O**) were observed in affected cells. After 12 h, disappearance of microtubules and abnormally condensed chromatin were common effects (**P**). **0210**: After 1 h of treatment, disoriented cortical microtubules (**Q**), along with many endoplasmic microtubules (**R**) could be observed in affected interphase cells. Other defects observed were the absence of perinuclear microtubules at preprophase (**S**, brackets indicate PPB) and altered phragmoplasts with abnormally thick cell plates (**T**). After 12 h, no microtubules could be detected, and chromatin state was also abnormal (**U**). **1810**: After 1 h of treatment, affected cells exhibited numerous endoplasmic microtubules (left cell in **V**), abnormally short microtubules attached to chromosomes (middle cell in **V**) and malformed mitotic spindles (right cell in **V**). Misaligned chromosomes, outside the equator plate (arrows in **W**) could also be observed, as well as masses of chromosomes attached to aberrant spindle-like microtubules (**X**,**Y**). After 12 h, no tubulin polymers could be detected, while chromatin was abnormally condensed (**Z**). Scale bars: 5 μm.

Microtubules appeared to be also affected by MC− extracts from certain non-MC-producing strains (Figure 12). Disorientation of cortical microtubules (Figure 12A,C,H,Q; cf. Figure 11A) and the formation of excess endoplasmic microtubules (Figure 12B,D,I,M,R, left cell in V; cf. Figure 11B) were common effects observed after treatment. Defects of the mitotic spindles (Figure 12E,K, right cell in V,W; cf. Figure 11D), lack of perinuclear microtubules in preprophase cells (Figure 12J,N,S; cf. Figure 11C, preprophase bands of microtubules are defined by brackets), and anomalies in phragmoplasts during cytokinesis (Figure 12F,G,L,O,T; cf. Figure 11F) were reported as well. For root cells treated with the 0499 and 0599 extracts, these effects were rather minor, compared to the control, detectable at all time points of exposure (1–24 h). However, the *Jaaginema* (0110 and 0210) extracts and the *Microcystis* 1810 extract induced such alterations after only 1 h of treatment, along with even harsher effects, including abnormal chromatin condensation (Figure 12V median cell, Figure 12X,Y). After longer exposure, affected cells appeared devoid of tubulin polymers, with abnormal chromatin condensation (Figure 12P,U,Z).

### 2.4. Induction of Oxidative Stress

**Figure 13:**
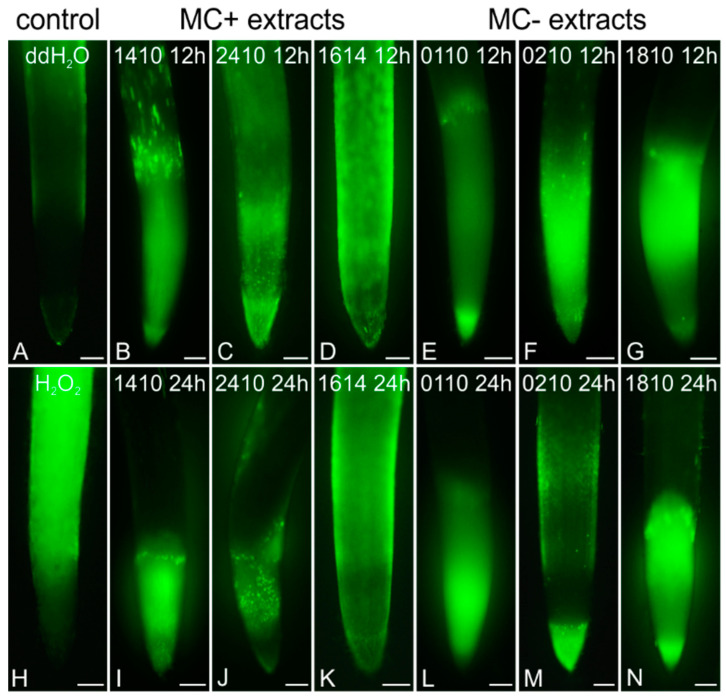
Imaging of H_2_O_2_ levels in root tips of *Oryza sativa*, after staining with 2,7-dichlorofluorescein diacetate (DCF-DA) of control (**A**,**H**) and extract-treated roots (**B**–**G**,**I**–**N**). Negative control roots were treated with double-distilled water (ddH_2_O_2_, **A**) for 24 h, while positive control roots were exposed to an aqueous solution of 10 mM H_2_O_2_ for 1 h (**H**). For extract-treated roots, strain codes and treatment durations are indicated on each image. Treatment with MC+ extracts (from MC-producing strains) for 12 h (**B**–**D**) and 24 h (**I**–**K**) led to increased concentrations of H_2_O_2_ in the root meristematic zone. Similar effects were also observed after 12 h (**E**–**G**) and 24 h (**L**–**N**) of treatment with MC− extracts from certain non-MC-producing strains (0110, 0210, 1810). Scale bars: 200 μm.

Six of the extracts induced oxidative stress in rice roots, compared to control (Figure 13A,H), initiating at 12 h of exposure. All *Microcystis* extracts, from both MC-producing (1410, 2410) and non-MC-producing (1810) strains, produced increased levels of H_2_O_2_ after 12 h (Figure 13B,C,G). After 24 h, fluorescence was only visible in cells of the root apical meristem (Figure 13I,J,N). MC− extracts from both *Jaaginema* strains (0110, 0210) also produced elevated H_2_O_2_ levels in treated roots after 12 h (Figure 13E,F) and 24 h (Figure 13L,M), while similar effects were observed after treatment with the MC+ extract of *Trichormus variabilis* TAU-MAC 1614 (Figure 13D,K).

## 3. Discussion

Almost all of the cyanobacterial extracts applied for treatments affected rice root cells. More specifically, all the MC+ extracts affected both F-actin (Figure 2, Figure 3 and Figure 10) and microtubules (Figure 11), and increased the levels of H_2_O_2_ (Figure 13), except *Raphidiopsis raciborskii* TAU-MAC 1414 extract, which did not induce an H_2_O_2_ increase. MC− extracts (from non-MC-producing strains), except *Nodularia* sp. TAU-MAC 0717 extract, caused F-actin disorders (Figure 4, Figure 5, Figure 6, Figure 7, Figure 8, Figure 9 and Figure 10). *Jaaginema* sp. TAU-MAC 0110 and 0210 and *Microcystis viridis* TAU-MAC 1810 extracts also affected microtubules and induced an increased H_2_O_2_ production (Figure 12 and Figure 13), while *Synechococcus* sp. TAU-MAC 0499 and *Chlorogloeopsis fritschii* TAU-MAC 0599 affected microtubules but did not increase H_2_O_2_ (Figure 12; for an overview, see Table 2). Therefore, it is further consolidated that extracts from various cyanobacterial strains (deriving from a multitude of environments; Table 3) target both plant microtubules and F-actin and are capable of inducing H_2_O_2_ production.

Many strains used in the current study have also been shown to have inhibitory effects on the growth of heterotrophic bacteria (namely: 0399, 0499, 0110, 0210, 1410, 1510, 1810, 2410) [29]. Additionally, extracts from the *Jaaginema* sp. TAU-MAC 0110 and 0210 strains have exhibited cytotoxic activity against human cancer cell lines, reportedly inducing F-actin alterations [29]. Their established bioactivity was further investigated by enzyme inhibition assays (Table 1).

The inhibitory effects of several cyanopeptides—beyond MCs—on various proteases [26] provide a useful tool for correlating the microscopically observed cytoskeletal changes with the activity of such compounds. Therefore, we demonstrated the effects of the extracts on hydrolytic enzymes. The inhibition of the activity of proteolytic enzymes, such as chymotrypsin, trypsin, elastase, and thrombin, by cyanobacterial extracts has been frequently reported [36,37]. Inhibition of both elastase and trypsin was also assigned to peptides such as micropeptins, cyanopeptolins, microviridins, and banyasides [37,38,39,40,41,42]. The production of potent inhibitors has also been found in several cyanobacteria, like *Microcystis*, *Planktothrix*, *Anabaena*, *Nostoc*, *Lyngbya,* and *Symploca* [43,44,45]. Inhibition of hydrolytic enzymes might not be a threat for extracellular enzymes, diluted in bulk water. However, the probable accumulation of cyanopeptides by aquatic organisms, including plants, may result in reaching an intracellular concentration high enough to inhibit intracellular enzymes, as plants cannot regulate their endogenous peptidase activity in combination with serine peptidases of cyanobacterial origin [46,47].

Cyanotoxins, such as MCs and cylindrospermopsin, are known to affect plant growth [48,49], cause chromatin defects [12], and induce disorganization of microtubules in plant cells [50,51]. MC-LR was recently found to induce F-actin alterations in *Oryza sativa* root cells [13]. However, even MC− extracts which did not affect microtubules, appeared to disrupt the F-actin network, implying that each cytoskeletal component is affected by independent mechanism of toxicity and suggesting that F-actin is a primary target of cyanobacterial toxicity, beyond MCs, in plant cells. To our best knowledge, this is the first report of cytoskeletal alterations in plant cells induced by extracts from cyanobacterial strains not producing MCs or cylindrospermopsin, underlining that several more cyanobacterial bioactive compounds are able to disrupt the plant cytoskeleton. The exact identity and mode of action of these compounds (which may also exert their effects synergistically) remain to be further studied.

Oxidative stress, detected in roots treated with extracts from certain strains (Figure 13), could also play a role in the induction of cytoskeletal defects. Elevated ROS levels have been associated with reorganization of microtubules in plant cells [52]. ROS-induced F-actin remodeling has also been reported in innate immunity responses of *Arabidopsis thaliana* pavement cells [53], suggesting that the increase in ROS levels due to extract treatment could affect F-actin as well. Nevertheless, cytoskeletal alterations were also observed after treatment with extracts that did not induce oxidative stress. In addition, increased ROS production was detected at 12 h, while disorders of the cytoskeleton appeared even after 1 h of treatment. This is possibly a hint that cyanobacterial toxicity against the plant cytoskeleton may not always involve ROS.

Cyanobacterial extracts induced a multitude of alterations in rice root cells and these findings could also be of ecological significance. Indeed, cyanobacteria produce a wide range of bioactive compounds [54,55] and cyanobacterial blooms often consist of several species [56,57,58]. An emerging challenge is to identify the above compounds and analyze their specific effects on plant cells. This would be the target of further research.

## 4. Materials and Methods

### 4.1. Culture of Cyanobacteria, Biomass Collection, and Preparation of Extracts

Sixteen cyanobacterial strains of the TAU-MAC culture collection [59], representatives of various taxonomic orders and habitats, were used for experimental purposes (Table 3).

**Table 3:** The cyanobacterial strains used here and relevant information. “+”: microcystins (MCs) detected. “−“: no MCs detected.

Taxonomic Order	Strain	Habitat	Lifestyle	Detection of MCs	References
Chroococcales	*Microcystis flos-aquae* TAU-MAC 1410	Freshwater	Planktic	+	[29,59]
	*Microcystis viridis* TAU-MAC 1810	Freshwater	Planktic	−	[29,59]
	*Microcystis* sp. TAU-MAC 2410	Freshwater	Planktic	+	[29,59]
Synechococcales	*Synechococcus* sp. TAU-MAC 0499	Freshwater	Planktic	−	[29,59]
	*Jaaginema* sp. TAU-MAC 0110	Freshwater	Planktic	−	[29,59]
	*Jaaginema* sp. TAU-MAC 0210	Freshwater	Planktic	−	[29,59]
Nostocales	*Calothrix epiphytica* TAU-MAC 0399	Freshwater	Epiphytic	−	[29,59]
	*Chlorogloeopsis fritschii* TAU-MAC 0599	Freshwater	Epiphytic	−	[29,59]
	*Raphidiopsis raciborskii* TAU-MAC 1414	Freshwater	Planktic	+ ^1^	[59,60]
	*Trichormus variabilis* TAU-MAC 1614	Freshwater	Planktic	+	Unpublished data
	*Nostoc oryzae* TAU-MAC 0315	Freshwater	Epiphytic	−	Unpublished data
	*Nodularia* sp. TAU-MAC 0717	Brackish	Planktic	−	Unpublished data
	*Scytonema* sp. TAU-MAC 1218	Terrestrial cave	Epilithic	−	Unpublished data
Oscillatoriales	*Planktothrix agardhii* TAU-MAC 0514	Freshwater	Planktic	−	[29,59]
	*Phormidium* sp. TAU-MAC 0517	Terrestrial cave	Epilithic	−	Unpublished data
	*Lyngbya* sp. TAU-MAC 4418	Intertidal zone	Epilithic	−	Unpublished data

^1^ Presence of MCs could not be unambiguously confirmed by LC–MS/MS analysis.

All strains were cultured in Erlenmeyer flasks using either BG-11 or MN (for marine or brackish strains) medium, with or without (for Nostocales strains) the addition of inorganic nitrogen [61] at 20 ± 1 °C in a 12:12 h light:dark cycle under white fluorescent lamps with a photon flux density of 10 μmol m^−2^ s^−1^ (for details on the media used, see Table A1).

Extracts from the cyanobacterial strains were prepared according to [13]. Wet biomass from each culture was harvested by centrifugation at the exponential growth phase (about 30 days) and freeze-dried. Lyophilized biomass (150 mg dry weight) from each strain was dissolved thrice in a total of 21 mL of 75% (*v*/*v*) methanol. All samples were sonicated during the first extraction step for 10 min. Methanol was finally evaporated and each pellet was resuspended in 5 mL of double-distilled water. Aqueous extracts were filtered through Whatman Polydisc TF filters (Whatman plc, Little Chalfont, UK) with a pore size of 0.2 μm.

### 4.2. Enzyme Inhibition Assays

Dilutions of crude cyanobacterial extracts (1:25 and 1:50) in double-distilled water (ddH_2_O) were used. All enzyme inhibitors were also diluted in ddH_2_O, at various concentrations. All assays were performed in 96-well microplates.

#### 4.2.1. Protein Phosphatase Inhibition Assay

Potential bioactivity of the cyanobacterial extracts against PP1 activity was tested using the colorimetric method assay protocol described in [62], modified according to [28,36]. PP1 (Santa Cruz Biotechnology, Dallas, TX, USA, 1.7 U mL^−1^) was diluted in buffer containing 50 mM Tris-HCl (pH 7.4), 1 mg mL^−1^ bovine serum albumin (BSA), 1 mM MnCl_2_, and 2 mM dithiothreitol (DTT). The substrate, *p*-nitrophenyl phosphate (*p*-NPP, 5.5 mg mL^−1^), was diluted in buffer containing 50 mM Tris-HCl (pH 8.1), 0.5 mg mL^−1^ BSA, 20 mM MgCl_2_ × 6H_2_O, and 0.2 mM MnCl_2_ × 4H_2_O. Microcystin-LR (Enzo Life Sciences, Farmingdale, NY, USA) was used as standard PP1 inhibitor. Then, 10 μL of either sample or inhibitor solution + 10 μL of enzyme solution were added in each well and the reaction started by adding 200 μL of *p*-NPP. Loaded microplates were incubated for 2 h at 37 °C and absorbance was measured with a microplate reader at 405 nm.

#### 4.2.2. Trypsin Inhibition Assay

Extracts were tested for trypsin inhibition using the assay described by [42], with modifications. Porcine trypsin (1 mg mL^−1^) and its substrate, *N*_α_-benzoyl-DL-arginine 4-nitroanilide hydrochloride (BAPNA, Santa Cruz Biotechnology, 2 mM), were diluted in buffer containing 50 mM Tris-HCl (pH 7.5), 100 mM NaCl, and 1 mM CaCl_2_. Aprotinin was used as enzyme inhibitor. A total of 10 μL of sample/inhibitor solution/ddH_2_O + 10 μL of enzyme + 100 μL of buffer were added in each well and preincubated for 5 min at 36 °C. Afterwards, 100 μL of substrate were loaded and the mixture was incubated for 20 min at the same temperature. Absorbance was measured at 405 nm.

#### 4.2.3. Elastase Inhibition Assay

Elastase inhibition was tested using the protocol by [63], modified according to [36]. Porcine elastase (75 μg mL^−1^) and its substrate, *N*-succinyl-Ala-Ala-Ala-p-nitroanilide (2 mM) were diluted in 0.2 M Tris-HCl (pH 8) buffer. Elastatinal was used as enzyme inhibitor. Then, 10 μL of sample/inhibitor solution/ddH_2_O + 10 μL of enzyme + 150 μL of buffer were added in each well and preincubated for 15 min. The reaction was started by adding 30 μL of substrate and the mixture was incubated for another 10 min. Absorbance was measured at 405 nm.

### 4.3. Plant Material and Treatments

Rice (*Oryza sativa* cv. Axios), generously provided by the National Cereal Institute (Thessaloniki, Greece), was germinated on moistened filter paper at 24 ± 1 °C in the dark. Four- to five-day-old seedlings were transferred in Eppendorf tubes containing either aqueous cyanobacterial extracts or double-distilled water (control) and placed with their roots submerged for various time periods (1, 12, or 24 h) under the same conditions as during germination. After treatment, root tips were prepared for fluorescence microscopy. All chemicals and reagents were purchased from Applichem (Darmstadt, Germany), Sigma-Aldrich (Taufkirchen, Germany), and Merck (Darmstadt, Germany) and all the following experimental procedures were performed at room temperature, unless otherwise stated.

### 4.4. Cytoskeletal Studies

#### 4.4.1. Tubulin Immunolabeling

For microtubule observations, control and extract-treated root rips were excised and fixed in 4% (*w*/*v*) paraformaldehyde (PFA) solution in PEM buffer (50 mM PIPES, 5 mM EGTA, 5 mM MgSO_4_, pH 6.8) + 5% (*v*/*v*) dimethyl sulfoxide (DMSO) for 1 h. Fixed specimens underwent cell wall digestion with a 3% (*w*/*v*) Macerozyme R-10 + 3% (*w*/*v*) cellulase Onozuka R-10 (Duchefa Biochemie, Haarlem, The Netherlands) solution in PEM, for 90 min. After digestion, root tips were squashed gently on coverslips coated with poly-l-lysine and the released cells were left to dry and adhere. Afterwards, they were extracted with a 5% (*v*/*v*) DMSO + 1% (*v*/*v*) Triton X-100 solution in phosphate-buffered saline (PBS, pH 7.2), for 1 h. Rat anti-*α*-tubulin (YOL 1/34, Bio-Rad Laboratories, Hercules, CA, USA or Santa Cruz Biotechnology, Dallas, TX, USA) was used as primary antibody (diluted 1:50 in PBS, incubated overnight) and anti-rat IgG Alexa Fluor 488 (Cell Signaling Technology, Danvers, MA, USA) as secondary antibody (diluted 1:300 in PBS, incubated at 37 °C for 2 h). DNA counterstaining was performed using 4′,6-diamidino-2-phenylindole (DAPI) for 5 min. Finally, specimens were mounted with anti-fade medium (PBS 1:2 glycerol (*v*/*v*) + 0.5% (*w*/*v*) *p*-phenylenediamine).

#### 4.4.2. F-actin Labeling

F-actin was labeled with fluorescent phalloidin, according to [64], with slight modifications. F-actin in rice root tips was prestabilized with 300 μM m-maleimidobenzoyl-*N*-hydroxysuccinimide ester in PEM + 0.1% (*v*/*v*) Triton X-100 for 30 min in the dark and fixed with 4% (*w*/*v*) PFA in PEM + 5% (*v*/*v*) DMSO + 0.1% (*v*/*v*) Triton X-100 + DyLight 554-phalloidin (Cell Signaling Technology, Danvers, MA, USA) 1:400 for better F-actin preservation. Fixed specimens were washed with PEM, extracted in 5% (*v*/*v*) DMSO + 1% (*v*/*v*) Triton X-100 in PBS for 1 h and incubated with DyLight 554-phalloidin (diluted 1:40 in PBS) at 37 °C for 2 h. DNA was counterstained with DAPI and all specimens were mounted with antifade medium.

#### 4.4.3. Confocal Fluorescence Microscopy

Cytoskeletal elements in fluorescent specimens were observed under a Zeiss Observer.Z1 (Carl Zeiss AG, Munich, Germany) microscope, equipped with the LSM780 confocal laser scanning (CLSM) module and the appropriate filters for each fluorophore. Imaging was achieved with ZEN2011 software, according to the manufacturer’s instructions.

#### 4.4.4. Fluorescence Intensity Measurements

Fluorescence intensity measurements of F-actin in control and extract-treated root tip cells (from the meristematic and differentiation zone) were performed in maximum intensity projections of serial CLSM sections with ImageJ (https://imagej.net/Fiji), according to [65]. The corrected total cell fluorescence (CTCF; [66]), was calculated with the formula: CTCF = integrated density − (area of selected cell × mean fluorescence of background readings). Thirty individual cells from three different roots per treatment were measured for fluorescence intensity and results were statistically analyzed (ANOVA with Dunnett’s test) with GraphPad Prism, at a significance of *p* < 0.05.

### 4.5. Detection of Hydrogen Peroxide Production

H_2_O_2_ was detected using 2,7-dichlorofluorescein diacetate (DCF-DA), according to [67,68]. Extract-treated rice seedlings were incubated with their roots submerged in 25 μM DCF-DA aqueous solution for 30 min, washed with double-distilled water, and examined under a Zeiss AxioImager.Z2 light microscope (Carl Zeiss AG, Munich, Germany), equipped with an AxioCam MRc5 camera (Carl Zeiss AG). Seedlings treated with double-distilled water +5% DMSO for 24 h or 10 mM H_2_O_2_ for 1 h, were used as positive and negative control, respectively. Images were captured using AxioVision Rel. 4.8.2 software (Carl Zeiss AG).

## 5. Conclusions

According to our observations, not only MC+ extracts, but also MC− extracts from various non-MC-producing cyanobacterial strains (isolated from a multitude of environments) were able to induce alterations in both plant cytoskeletal components, i.e., F-actin and microtubules, in *Oryza sativa* (rice) root cells (Table 2). This is the first report of cyanobacterial extracts, not containing any known cyanotoxins, affecting the plant cytoskeleton. Therefore, it is supported that MCs or cylindrospermopsin are not the only cyanobacterial compounds able to induce cytoskeletal alterations in plant cells. Certain, but not all, MC− extracts also raised H_2_O_2_ levels in treated roots, which is an implication that oxidative stress is not necessarily involved in the cytoskeletal alterations observed. Obviously, several bioactive compounds may be present in the extracts of non-MC-producing cyanobacterial strains, the identity and mode of action of which remains to be revealed by future studies.

## Figures and Tables

**Table 1:** Inhibitory effects of the cyanobacterial extracts on PP1, trypsin, and elastase, with reference to each strain. “+”: inhibition; “−“: no effect.

MCs Production Status	TAU-MAC Strain	PP1 ^1^	Trypsin ^1^	Elastase ^2^
MC-producing	*Microcystis flos-aquae* 1410	+	−	+
	*Microcystis* sp. 2410	+	−	+
	*Trichormus variabilis* 1614	+	+	+
	*Raphidiopsis raciborskii* 1414 ^3^	−	−	+
Non-MC-producing	*Jaaginema* sp. 0110	−	−	+
	*Jaaginema* sp. 0210	−	−	+
	*Microcystis viridis* 1810	−	−	+
	*Synechococcus* sp. 0499	−	−	+
	*Chlorogloeopsis fritschii* 0599	−	−	+
	*Calothrix epiphytica* 0399	−	−	+
	*Planktothrix agardhii* 0514	−	−	+
	*Phormidium* sp. 0517	−	−	+
	*Scytonema* sp. 1218	−	−	+
	*Nostoc oryzae* 0315	−	−	+
	*Lyngbya* sp. 4418	−	−	+
	*Nodularia* sp. 0717	−	−	−

^1^ Inhibition reported at 1:25 and 1:50 dilutions. ^2^ Inhibition reported at 1:25 dilution. ^3^ Presence of MCs could not be unambiguously confirmed by LC–MS/MS analysis.

**Table 2:** Effects observed in rice root cells after exposure to cyanobacterial extracts, with reference to each strain. “✓”: affected; “✘”: no effect.

MCs Production Status	TAU-MAC Strain	F-actin	Microtubules	H_2_O_2_ Levels
MC-producing	*Microcystis flos-aquae* 1410	✓	✓	✓
	*Microcystis* sp. 2410	✓	✓	✓
	*Trichormus variabilis* 1614	✓	✓	✓
	*Raphidiopsis raciborskii* 1414 ^1^	✓	✓ ^3^	✘
Non-MC-producing	*Jaaginema* sp. 0110	✓	✓	✓
	*Jaaginema* sp. 0210	✓	✓	✓
	*Microcystis viridis* 1810	✓	✓	✓
	*Synechococcus* sp. 0499	✓	✓ ^3^	✘
	*Chlorogloeopsis fritschii* 0599	✓	✓ ^3^	✘
	*Planktothrix agardhii* 0514	✓	✓ ^3,4^	✘
	*Calothrix epiphytica* 0399	✓	✘	✘
	*Phormidium* sp. 0517	✓	✘	✘
	*Scytonema* sp. 1218	✓	✘	✘
	*Nostoc oryzae* 0315	✓ ^2^	✘	✘
	*Lyngbya* sp. 4418	✓ ^2^	✘	✘
	*Nodularia* sp. 0717	✘	✘	✘

^1^ Presence of MCs could not be unambiguously confirmed by LC–MS/MS analysis. ^2^ F-actin exhibited minor abnormalities. ^3^ Microtubules exhibited minor abnormalities. ^4^ Images not shown.

## Abbreviations

CLSM	Confocal laser scanning microscope
CTCF	Corrected total cell fluorescence
DAPI	4′,6-diamidino-2-phenylindole
DCF-DA	2,7-dichlorofluorescein diacetate
DMSO	Dimethyl sulfoxide
LC–MS/MS	Liquid chromatography with tandem mass spectrometry
MC	Microcystin
MC+	Microcystin-rich
MC−	Microcystin-devoid
PBS	Phosphate buffer saline
PEM	PIPES EGTA MgSO_4_
PFA	Paraformaldehyde
PPB	Preprophase band
PP1	Protein phosphatase 1
PP2A	Protein phosphatase 2A
ROS	Reactive oxygen species

## Appendix A

**Table A1:** The studied cyanobacterial strains: relevant information and media used for culture. “BG-11” and “MN”: contains NaNO_3_. “BG-11^0^” and “MN^0^”: no NaNO_3_ added. For MN medium, 75% (*v*/*v*) seawater was used, according to references.

Taxonomic Order	Strain	Culture Medium	References
Chroococcales	*Microcystis flos-aquae* TAU-MAC 1410	BG-11	[61,69]
	*Microcystis viridis* TAU-MAC 1810	BG-11	
	*Microcystis* sp. TAU-MAC 2410	BG-11	
Synechococcales	*Synechococcus* sp. TAU-MAC 0499	BG-11	
	*Jaaginema* sp. TAU-MAC 0110	BG-11	
	*Jaaginema* sp. TAU-MAC 0210	BG-11	
Nostocales	*Calothrix epiphytica* TAU-MAC 0399	BG-11^0^	
	*Chlorogloeopsis fritschii* TAU-MAC 0599	BG-11^0^	
	*Raphidiopsis raciborskii* TAU-MAC 1414	BG-11^0^	
	*Trichormus variabilis* TAU-MAC 1614	BG-11^0^	
	*Nostoc oryzae* TAU-MAC 0315	BG-11^0^	
	*Nodularia* sp. TAU-MAC 0717	MN^0^	
	*Scytonema* sp. TAU-MAC 1218	BG-11^0^	
Oscillatoriales	*Planktothrix agardhii* TAU-MAC 0514	BG-11	
	*Phormidium* sp. TAU-MAC 0517	BG-11	
	*Lyngbya* sp. TAU-MAC 4418	MN	
